# Carbon-Based Nanomaterials Decorated Electrospun Nanofibers in Biosensors: A Review

**DOI:** 10.1021/acsomega.3c00798

**Published:** 2023-12-18

**Authors:** Nur Melis Kilic, Sultan Sacide Gelen, Simge Er Zeybekler, Dilek Odaci

**Affiliations:** † Ege University, Faculty of Science Biochemistry Department, 35100 Bornova-Izmir, Turkey

## Abstract

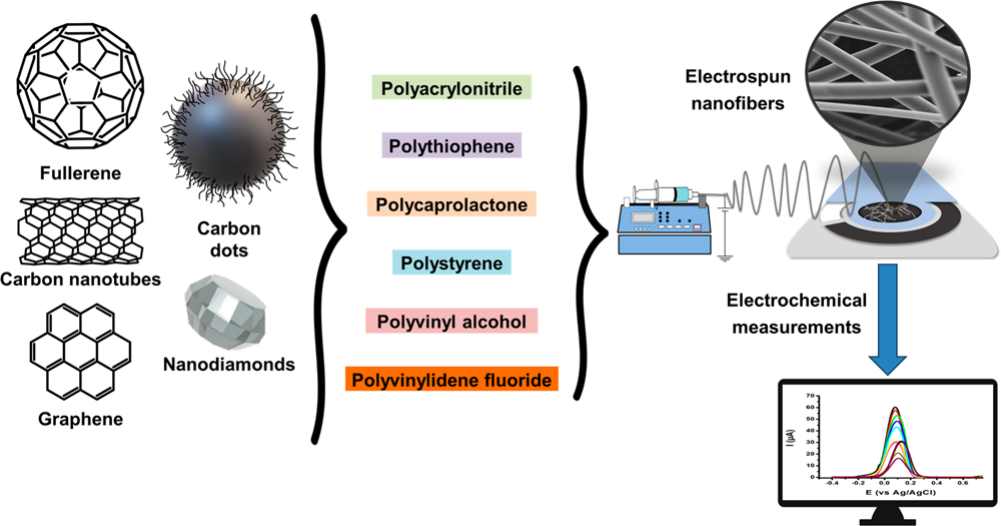

Nanomaterials have revolutionized scientific research due to their exceptional physical and chemical capabilities. Carbon-based nanomaterials such as graphene and its derivates have excellent electrical, optical, thermal, physical, and chemical properties that have made them indispensable in several industries worldwide, including medicine, electronics, and energy. By incorporating carbon-based nanomaterials as nanofillers in electrospun nanofibers (ESNFs), smoother and highly conductive nanofibers can be achieved that possess a large surface area and porosity. This approach provides a superior alternative to traditional materials in the development of improved biosensors. Carbon-based ESNFs, among the most exciting new-generation materials, have many applications, including filtration, pharmaceuticals, biosensors, and membranes. The electrospinning technique is a highly efficient and cost-effective method for producing desired nanofibers compared to other methods. Various types of natural and synthetic organic polymers have been successfully utilized in solution electrospinning to produce nanofibers directly. To create diagnostics devices, various biomolecules like antibodies, enzymes, aptamers, ligands, and even cells can be bound to the surface of nanofibers. Electrospun nanofibers can serve as an immobilization matrix to create a biofunctional surface. Thus, biosensors with desired features can be produced in this way. This study comprehensively reviews biosensors that integrate nanodiamonds, fullerenes, carbon nanotubes, graphene oxide, and carbon dots into electrospun nanofibers.

## 1 Electrospun Nanofibers (ESNFs)

An adaptable and practical method for creating ultrathin fibers is electrospinning.^1^ In electrospinning, a polymer melt or the solution is transformed into fibers by a powerful electric field.^2^ The polymer droplet trapped at the nozzle by surface tension will build up charges on the surface caused by the external electric field and experience an electric field that is the opposite of the surface tension. The droplet at the nozzle elongates from a spherical shape to a cone shape, generating a “Taylor cone,” as the electric field is steadily raised. The charged solution will be propelled from the tip of the Taylor cone to form a jet when the electric field strength reaches a critical point, where it will defeat the liquid’s surface tension. Through the processes of solution volatilization and fiber solidification, the jet passes through the atmosphere and deposits on the collector to create fibrous films.^3,4^

**Figure 1:**
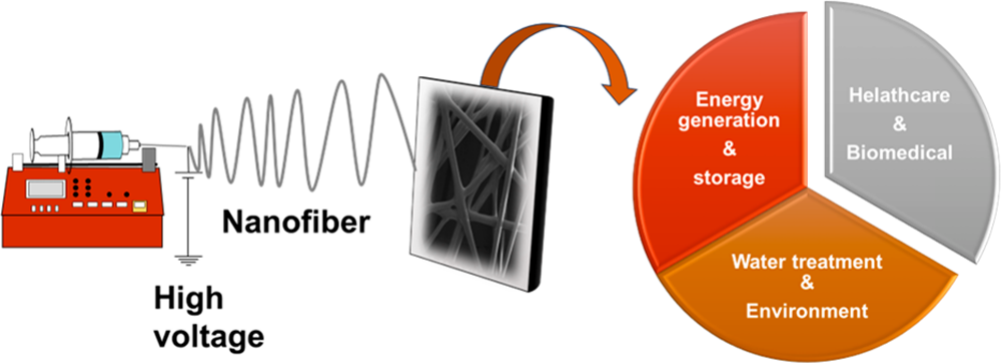
Schematic illustration of electrospinning and examples of applications of ESNFs.

The three main categories of components that influence the production of nanofibers by electrospinning are solution parameters, electrospinning parameters, and ambient parameters. In addition to the concentration, molecular weight, and relative molecular mass distribution of the polymer, the solution parameters also include the surface tension, type of solvent, and conductivity of the solution. Electrospinning process parameters comprise the applied voltage, fluid flow rate, collector-polymer distance, and needle diameter. The three main elements of the environmental parameters are temperature, humidity, and airflow.^5^ All these variables change depending on the polymer used and enable the production of highly porous, homogeneous, and smooth nanofibers from the beaded fiber. The performance of continuous ESNFs can be enhanced by selecting a solvent based on surface tension or by incorporating appropriate surfactants.^6^Figure 1 provides a representation of electrospinning as well as some applications of ESNFs.

**Figure 2:**
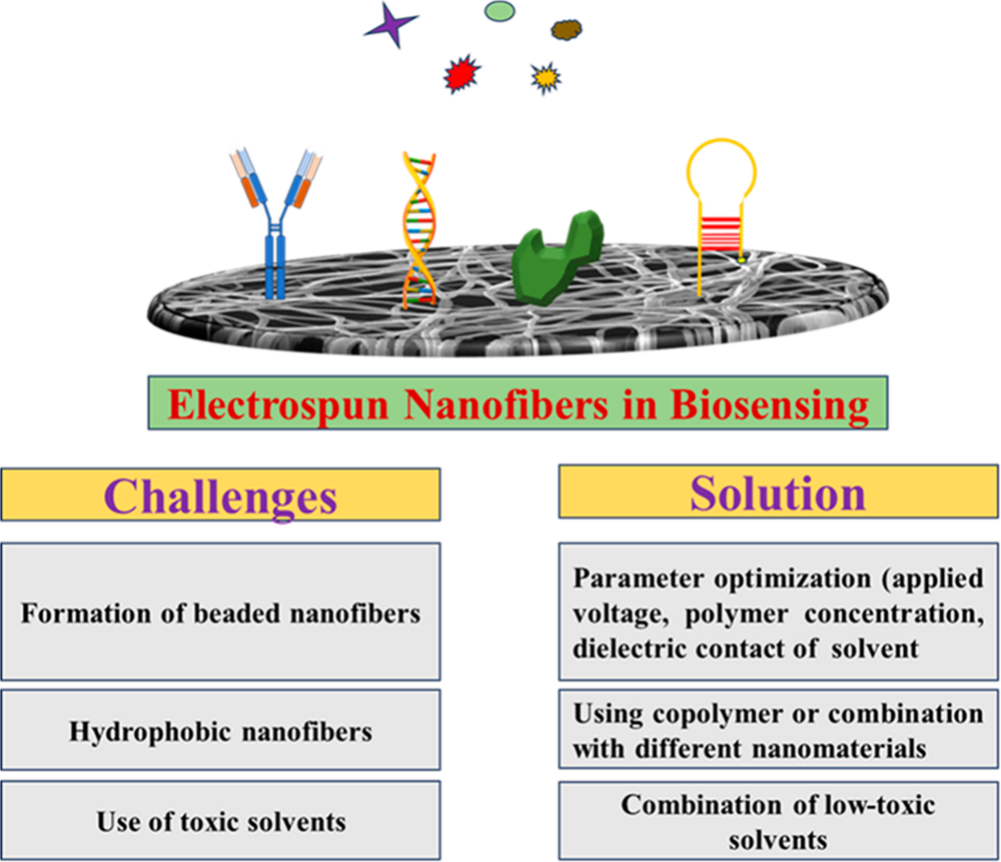
Challenges and solutions of ESNFs in biosensor systems.

There are several fabrication techniques such as melt spinning,^7^ solution spinning,^8^ and melt blowing^9^ to create nanofibers; however, the electrospinning technique is the most popular way to produce elongated, smooth, and uniform fibers with the desired nano/microscale.^10,11^ Moreover, this technique is a very uncomplicated and low-priced method. ESNFs can be used in various applications such as biomedical,^12^ drug delivery systems,^13^ environmental, water treatment (affinity membranes),^14^ and electromagnetic interference (EMI) shielding.^15,16^ The fibers, with their high surface area and porosity properties,^21^ can also be used in industrial applications such as food packaging,^17^ energy storage/conversion,^18−20^ and sensors.^22^ Additionally, ESNFs are flexible nanomaterials suitable for catalysts in air electrodes.^23^ Notably, ESNFs are among the more promising nanomaterials for sensor applications due to their huge surface areas. ESNFs can be produced with different structures such as spring/helical ESNFs, porous ESNFs, core–shell ESNFs, hollow electrospun fibers, and triaxial ESNFs,^24^ and additional analytes are allowed to adhere to the sensor surface which boosts sensitivity. Additionally, the sensor’s conductivity is greatly increased by nanofibers modified with high electrical conductivity doping agents. For the creation of clinical diagnostics devices, various biomolecules like antibodies, enzymes, aptamers, ligands, and even cells can be bound to the surface of nanofibers (biosensors). ESNFs can serve as an immobilization matrix to create a biofunctional surface. Combining electrospinning with novel materials may benefit the production of smart fibers that respond to pH, light, electric field, and magnetic field using responsive polymers.^25^ Furthermore, the highly porous structure of nanofibers supplies advanced catalytic efficiency due to low mass transfer resistance.^26,27^ ESNFs in biosensor systems have many advantages; including adjustable size (micro to nano), a large surface area, biocompatibility, suitability as a good immobilization matrix, cost-effectiveness, and the ability to be decorated with other nanomaterials.^28^ However, they also come with some limitations, such as hydrophobicity, the use of toxic solvents, and the formation of beaded nanofibers. Figure 2 shows these limitations and solutions.

Various natural and synthetic organic polymers, totaling over 100, have been successfully utilized in solution electrospinning to produce nanofibers directly. Polystyrene (PS) and polyvinyl chloride (PVC) nanofibers are produced for environmental protection.^29,30^ A variety of biocompatible and biodegradable synthetic polymers, including poly(ε-caprolactone) (PCL), poly(lactic acid) (PLA), and poly(lactic-*co*-glycolic acid) (PLGA), are used for creating ESNFs as scaffolds in biomedical fields.^31−33^ Nanofibers have been produced from natural biopolymers, such as DNA, silk fibroin, fibrinogens, dextran, chitin, chitosan, alginate, collagen, and gelatin. Electrospinning of conductive polymers like polyaniline (PANi) and polypyrrole (PPy) has facilitated the creation of nanofibers.^34,35^ This process has proved to be highly effective in producing conductive nanofibers with excellent properties. Poly(vinylidene fluoride) (PVDF), a functional polymer, can be used to produce nanofibers for piezoelectric/pyroelectric uses.^3,36^Table 1 offers a concise and informative summary of spinnable polymers and their applications in biosensors.

**Table 1:** Spinnable Polymers and Biosensor Applications^a^

nanomaterials	techniques	analyte	ref
PS-PAMAM/AOX	conductometric	MeOH	(37)
PCL-PPY-MWCNT/TNF-α-antibody	electrochemical	TNF-α	(38)
CA-CS/GOx	electrochemical	glucose	(39)
PVDF/PEI/anti-METH	electrochemical	METH	(40)
PCL/PAA/anti-CRP	fluorescence	CRP	(41)
PS/rGO-MNP-PDA/anti-CRP	electrochemical	CRP	(21)
PS/GO-APTES/anti-CD36	electrochemical	CD36	(42)
PVA/PEI/AuNPs/GOx	electrochemical	glucose	(43)
PVA/chitosan/ChOx	colorimetric	cholesterol	(44)
PASP	colorimetric	Cu^2+^ or Fe^3+^	(45)
PA6/PAH-MWCNT	electrochemical	dopamine	(46)
polyacrylonitrile/zein-rGO/anti-IGF-1	electrochemical	IGF-1	(47)

a PS-PAMAM, polystyrene-polyamidoamine dendrimer; MeOH, methanol; AOX, alcohol oxidase; PCL-PPY-MWCNT, polycabrolactone/polypyrrole/multiwalled carbon nanotube; TNF-α, tumor necrosis factor-alpha; CA-CS, cellulose acetate-chitosan; Gox, glucose oxidase; PVDF–PEI, poly(vinylidene fluoride)-polyethylenimine; anti-METH, antimethamphetamine antibody; METH, methamphetamine; PCL-PAA, polycabrolactone-poly(amic)acid; CRP, C-reactive protein; PS/rGO-MNP-PDA, polystyrene-graphene oxide/magnetic nanoparticles/polydopamine; PS/GO-APTES, polystyrene-graphene oxide-(3-aminopropyl)triethoxysilane; Au NPs/PVA/PEI, gold nanoparticles/poly(vinyl alcohol)/poly(ethyleneimine); ChOx, cholesterol oxidase; PASP, poly(aspartic acid); PA6/PAH, polyamide 6/poly(allylamine hydrochloride); polyacrylonitrile/zein-rGO, polyacrylonitrile (PAN)/zein-reduced graphene oxide; IGF-1, insulin-like growth factor-1.

Due to their superior conductivity, sizable surface area, cheap cost, and improved physicochemical features, carbon materials such as graphene, fullerene, and carbon nanotubes have been widely exploited in the creation of EM (electromagnetic) wave absorption materials. Nowadays, because of the properties of carbon and its huge area of use, nanofibers are used by researchers in many nanobiotechnological areas. Notably, nanofibers consisting of carbon-based nanomaterials could provide great opportunities to produce novel, ultrasensitive, and low detection limits sensing platforms such as electrochemical sensors, lab-on-chip devices, and wearable electronics with enhanced performance due to their superior electrical properties.^48^

**Figure 3:**
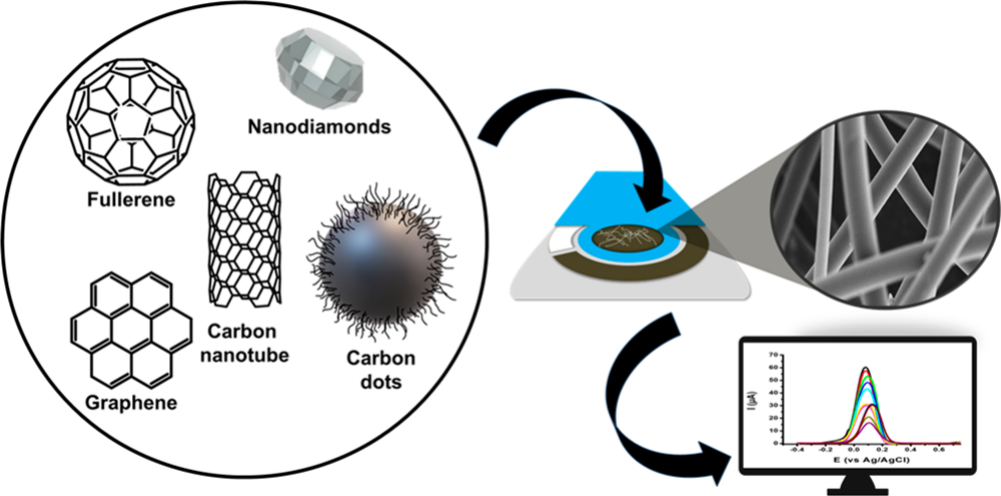
Carbon nanomaterial incorporated ESNFs production and application in biosensors.

This review presents the integration of various carbon-based nanomaterials into ESNFs. In addition, the mechanical, thermal, and electrical properties of carbon-based electrospinning nanofibers were evaluated comparatively, and the critical role of these properties was evaluated in the development of sensing platforms. Figure 3 displays a representation of carbon nanomaterial incorporated ESNFs production and application in biosensors.

### 1.1 Carbon Nanotube Incorporated ESNFs

**Figure 4:**
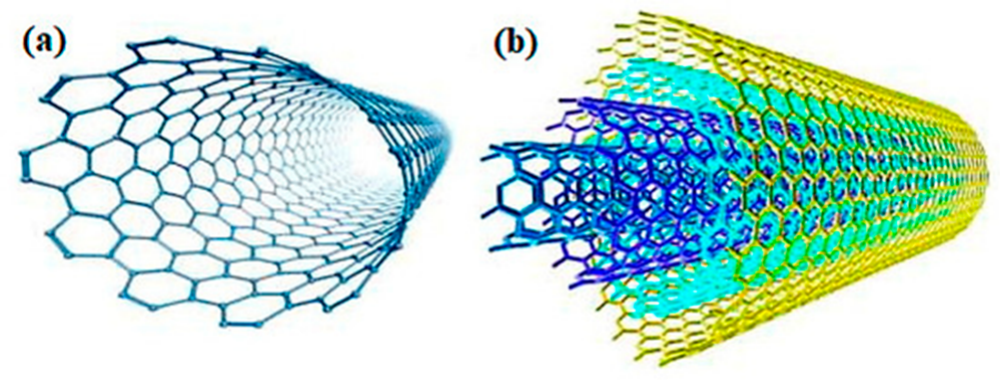
(a) Single-wall and (b) multiwall carbon nanotube.^54^

The quick development of nanobiotechnology, polymer chemistry, and semiconductor nanomaterial technology provides many new possibilities in sensor systems.^49^ Among carbon-based materials, carbon nanotubes (CNTs) have become increasingly popular in biosensor design thanks to excellent conductivity, and chemical mechanical and structural superiority.^50,51^ Discovered by Dr. Iijima in 1991, CNTs are cylindrical carbon-based nanomaterials consisting of a folded sheet of graphene.^52^ Structurally the most important CNTs are single-walled nanotubes (SWNTs) and multiwalled nanotubes (MWNTs). SWNT has only one layer of wrapped graphene. MWCNTs consist of several concentrically intertwined graphene helices with an interlayer spacing of 3.4 Å.^53^ A schematic illustration of single and multiwall carbon nanotubes is given in Figure 4.

Both types of CNTs appear to show high performance in nanosensor systems. In addition, CNTs with electrical conductivity are a suitable immobilization matrix for biomolecules such as aptamers, DNA, antibodies, enzymes, and small molecules because of their high surface areas.^55^ These properties of CNTs create a synergistic effect by allowing them to have low detection limits and high sensitivity in biosensor systems.^56,57^ Due to all the properties of CNTs, they have become the focus for many biosensor designs, from amperometric enzyme nanosensors to DNA hybridization biosensors.^50^ CNT-based nanosensors can be divided into electrochemical nanosensors, optical nanosensors, calorimetric nanosensors and other.^58^ To benefit from the properties of these nanomaterials with high efficiency, the CNT must be properly functionalized and immobilized.

**Figure 5:**
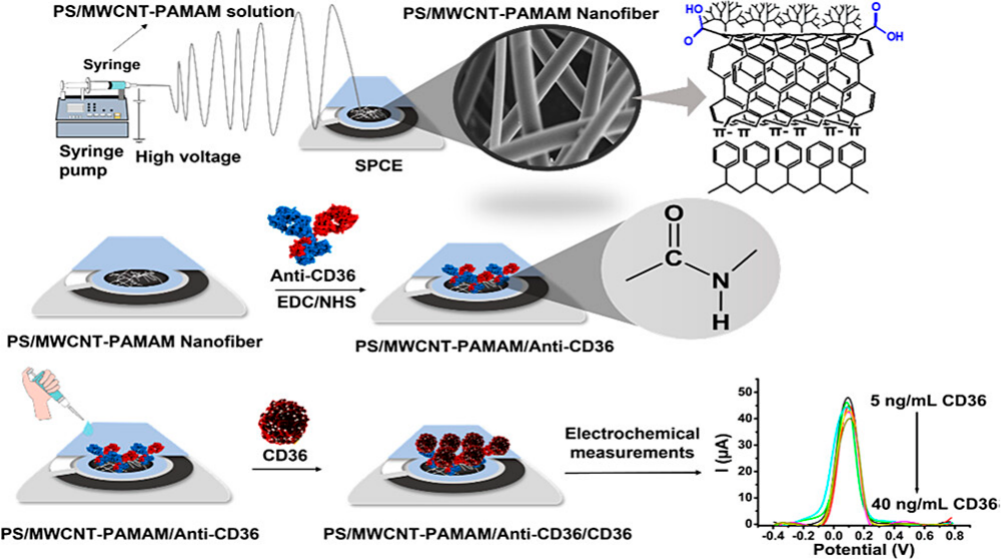
Schematic representation of PS/MWCNT-PAMAM ESNFs for its preparation and application.^67^

Currently, ESNFs are promising materials for the development of nanobiosensor systems, as they have excellent electrical conductivity, unique porous structures, large surface area, biocompatibility, and good stability. They are also inexpensive and effective.^59,60^ These properties of ESNFs give them great specificity and sensitivity with fast responsive reactions for real-time detection.^61^ By adding a small amount of CNTs to ESNFs, the strength, electrical conductivity, and thermal resistance of the final ESNFs-CNT composites appear to show superior properties compared to pristine polymeric ESNFs.^59^ The delocalized π electrons in the benzene rings endow these nanocomposites with high electrical conductivity,^62^ Thus, the idea of dispersing and aligning CNTs in an ESNFs matrix is promising for the use of ESFNs-CNT-based nanocomposite materials in biosensor systems.^63,64^ The use of ESFNs-CNT nanocomposites as an immobilization matrix with biomolecules to improve the electrochemical properties of biosensors has been reported in the literature.^65,66^ Zeybekler and Odaci developed an electrochemical biosensor system for the detection of the CD36 biomarker, which is an important biomarker in the early detection of atherosclerosis and diabetes. As seen in Figure 5, they modified polyamidoamine generation 3 (PAMAM G3) with oxidized MWCNT in this biosensor system. They added this MWCNT-PAMAM nanocomposite they synthesized to 35% polystyrene polymer solution. They obtained PS/MWCNT-PAMAM ESNFs. They tested the applicability of the PS/MWCNT-PAMAM they developed in the electrochemical biosensor system for CD36 determination. They reported that the linear determination range of this system was 5 to 40 ng/mL and the detection limit was 3.94 ng/mL.^67^Table 2 offers a concise and informative summary of recently developed biosensor systems based on ESNFs-CNTs.

**Table 2:** Recently Developed Biosensor Systems Based on ESNFs-CNTs^a^

CNTs-ESNFs	technique	analyte	ref
PABA/f-CNTs	electrochemical	glucose	(68)
PCL-PPY-MWCNT	electrochemical	TNF-α	(69)
PAN-MWCNTs/PEDOT and PAN-MWCNTs/PPy	electrochemical	glucose	(70)
PVA-NFs/MWCNTs/AuNPs	electrochemical	MUC_1_	(71)
CNT/Cys/Au/PAN ECNFs	electrochemical	Her-2	(72)
PA6/PAH/MWCNTs	electrochemical	dopamine	(46)
PBAT/PLA/MWCNTs	electrochemical	metronidazole	(73)
SU-8/MWCNTs	electrochemical	myoglobin (Myo),	(74)
		cardiac Troponin I (cTnI),	
		creatine kinase MB (CK-MB)	
PVA-SbQ/MWCNT-COOH	electrochemical	glucose	(75)
MWCNT@FC	electrochemical	uric acid	(76)
PA6/PAH/MWCNTs/AuNPs	electrochemical	CA19–9 (Pancreatic cancer)	(77)
PS/CMWCNT/PEG	electrochemical	cardiac Troponin I (cTnI)	(78)
CA/ZIF- 8@MWCNTs/AuNPs	electrochemical	glucose	(79)

a PABA/f-CNTs, poly(3-amino benzylamine)/functionalized multiwalled carbon nanotubes; PCL–PPY-MWCNT, polycaprolactone/polypyrol/multiwalled carbon nanotubes; CNT/Cys/Au/PAN-ECNF, carbon nanotubes/cysteamine/gold nanoparticles/polyacrylonitrile electrospun carbon nanofiber; PA6/PAH/MWCNTs, polyamide 6/poly(allylaminehydrochloride)/MWCNTs; PBAT/PLA/MWCNTs (poly(butylene adipate coterephthalate))/(poly(lactic acid)/ MWCNTs; PVA-SbQ/MWCNT-COOH, poly(vinyl alcohol) styrylpyridinium/MWCNT-COOH; MWCNT@FC, ferric ceria/MWCNT nanofibers; PA6/PAH/MWCNTs/AuNPs, polyamide 6 and poly(allylamine hydrochloride) ; PS/CMWCNT/PEG, polystyrene/carboxylated multiwalled carbon nanotube/polyethylene glycol; CA/ZIF- 8@MWCNTs/AuNPs, cellulose acetate/zeolitik imidazolat/MWCNTs/AuNPs.

As seen in the literature, biosensor systems developed with ESNFs-CNTs nanocomposites have been reported to provide good stability and high electron transfer capability. This suggests that biosensor systems developed based on small ESFNs-CNTs have promising potential for the detection of many analytes.

### 1.2 Graphene Oxide Incorporated ESNFs

**Figure 6:**
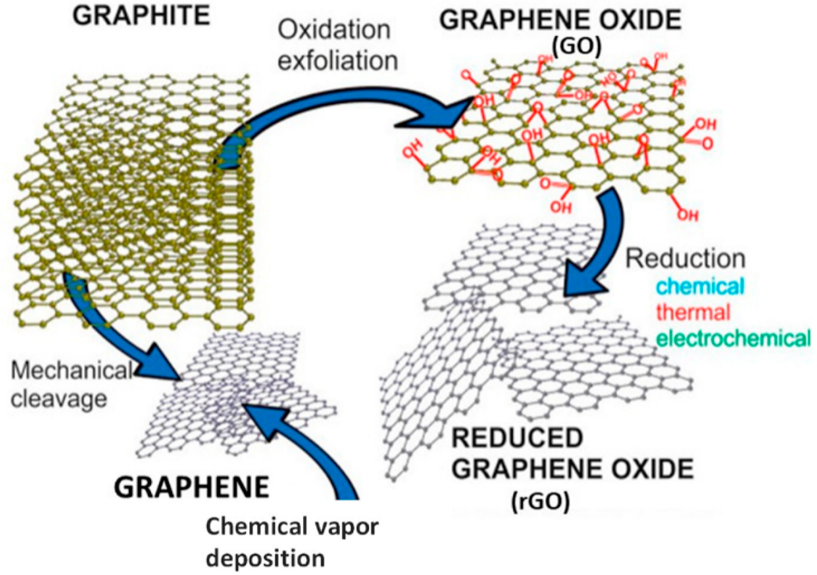
Schematic representation of graphene oxidation and reduction steps.^88^

Graphene (GR) is a two-dimensional (2D) nanomaterial that consists of a sp^2^ hybridized single-atom-thick sheet of carbon atoms and displays a honeycomb structure that can be converted into 0D, 1D, and 3D forms.^80,81^ Applications of GR in biosensors, biomedical applications, capacitors, and electromagnetic interference (EMI) shielding devices are increasing gradually thanks to high electrical conductivity, photocatalytic activity, antibacterial properties, and improved mechanical and thermal stability.^82−85^ Thus, GR attracts more attention than other carbon allotropes (carbon nanotubes or fullerenes). Additionally, the specific surface area of GR is about 2630 m^2^/g which provides high adsorption capacity for biosensor applications.^86^ However, it also has disadvantages such as a lack of band gap and poor water solubility. This situation greatly reduces its applicability in some fields.^87^ The synthesis of graphene derivatives can eliminate these disadvantages. For example, GO can be synthesized by functionalizing graphite layers with carboxyl, epoxy, and hydroxyl groups using strong oxidizing agents or exfoliation of graphite.^88^ These functional groups impart a hydrophilic character to GO, making it have excellent dispersibility in water or other polar solvents. Moreover, the functional groups offer reactive sites for GO functionalization with various modification agents.^89^ Modifying GO with modification agents via covalent or noncovalent bonds can inhibit aggregation and enable obtaining a stable dispersion.^90^ Furthermore, this modification can be specially designed to increase the adhesion/interaction of the GR sheets with the polymer matrix.^91^ In general, the surface modification of graphene can be performed in two ways: (i) noncovalent adsorption through secondary interactions such as H-bond, π–π interaction, hydrophobic, and van der Waals interactions, (ii) covalent bonds (C–C) can be formed between organic molecules and specific functional groups such as carboxyl (−COOH), hydroxyl (−OH), or epoxy found at the basal planes and edges of graphene layers.^92^ However, its electrical conductivity is relatively low compared to graphene since adding functional groups to the structure prevents the delocalization of π electrons in the benzene ring after oxidation of graphene. For this reason, it is not preferred much in the electrochemical field. However, reduced GO (rGO) can be obtained via reducing GO using chemical, thermal, or electrochemical reduction methods to obtain π-conjugation-rich graphene. Thus, π-conjugation in graphene sheets resembles pristine graphene and regains the conductivity of graphene.^89,93^ Graphene oxidation and reduction steps are given in Figure 6.

Three-dimensional (3D) graphene is also being used in electrochemical sensors since 3D macroporous structures (micro- (<2 nm), meso-sized (2–50 nm) pores and macro-sized (>50 nm) pores) can provide a wider adsorption area between the electrolyte and the electrode with large surface area and electrically conductive channels.^94^ Thus, it exhibits superior bioelectrochemical performance and supercapacitor properties.^95^ In general, 3D graphene can be obtained via 3D graphene oxide (3D-GO) reduction or with the use of gelation technologies with 2D-rGO sheets.^96,97^ However, electrochemical deposition synthesis can be used as a novel method to obtain 3D graphene on the electrode surface.^94^ Moreover, the functionalization of 3D graphene can be performed simply with various metal oxides or polymers.^98^ Despite the application potential of nanocomposites obtained by combining GO-based nanomaterial (GONM) and polymers, various problems may be encountered, such as the reduction of electrical or mechanical properties. Notably, graphene sheets aggregation in the polymer solution can occur due to intermolecular π–π interactions and van der Waals forces when conventional nanocomposite synthesis methods are used (solvent processing, in situ polymerization, etc.).^99,100^ The electrospinning technique which is an electrohydrodynamic process can be used to overcome these problems.^101,102^ Electrospinning offers an easy and effective way of integrating GONM into the structures of polymers. This technique converts GO layers with extremely high aspect ratios in the polymer solution into nanosized fibers instead of continuous sheets. Therefore, the agglomeration problem is eliminated, and the exfoliated GO exhibits better dispersion.^103^ Typically, the nanomaterial made from a graphene derivative is added to the polymer melt after electrospinning. Moreover, high-temperature or chemical reduction techniques can be used to produce rGO nanofibers.^104^

**Figure 7:**
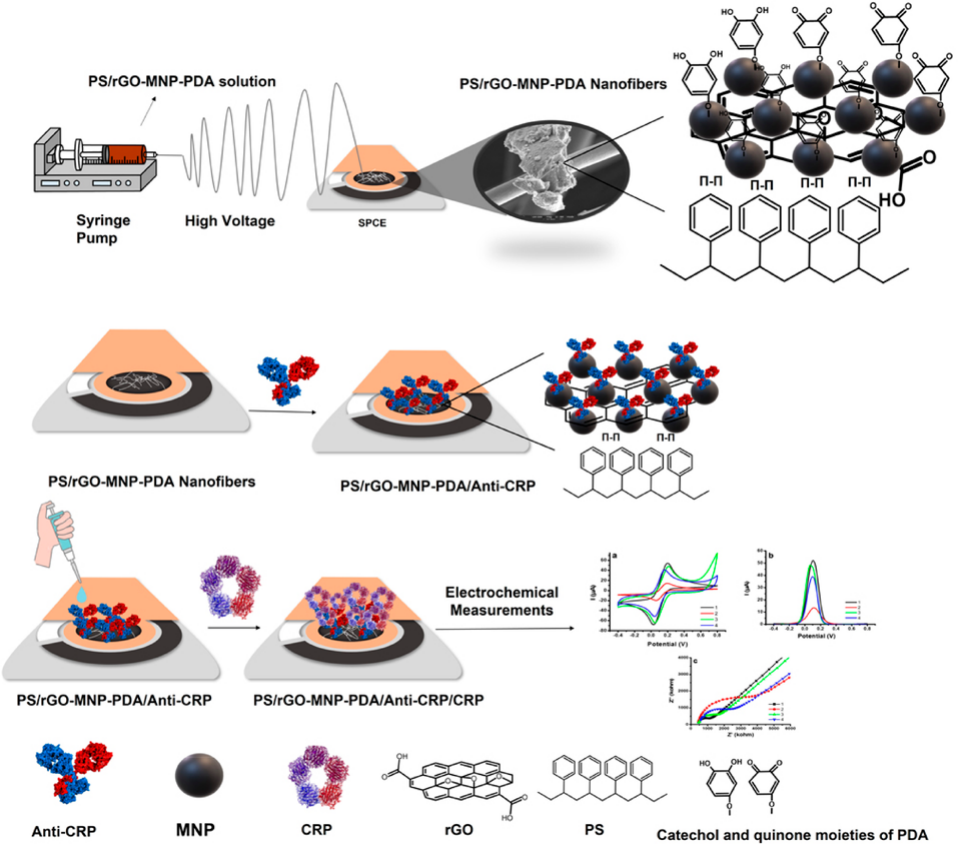
Schematic representation of the preparation of PS/rGO-MNP-PDA/anti-CRP modified SPCE and electrochemical detection of CRP.^21^

The use of GONM in electrospinning as nanofillers allows the production of nanofibers with desired properties (nanofiber diameter, mechanical properties, conductivity, or porosity). Nanofiller properties can also be adjusted by optimizing the electrospinning parameters and solution parameters.^105^ It has been reported in the literature that the nanofibers obtained by combining graphene with synthetic or natural polymers in the electrospinning process and using it as a nanofiller material exhibit remarkable properties such as conductivity, hydrophilicity, and chemical stability.^21,42,106,107^ For example, Zhou et al. applied in situ polymerization, electrospinning, and in situ thermal conversion to obtain polyimide/rGO (PI/rGO) nanofibers. They found that the in situ strategies used in this study helped distribute rGO in individual ESNFs and improved the interaction between rGO and PI. Furthermore, the PI/rGO composite nanofibers exhibited exceptional thermal stability, with a glass transition temperature (*T*_g_) of over 295 °C and a 5% thermal decomposition temperature (T_5%_) of over 539 °C.^108^ Furthermore, the functionalization of ESNFs with GONM provides highly active reaction regions on the electrode surface for the immobilization of various biological molecules. Thus, optical sensor/immunosensor/aptasensor/enzymatic sensor platforms can be produced with better biosensing performance.^109^ Moreover, GONM can be modified with various metal oxides during electrospinning or wet chemical processing. Thus, improved functionality and distribution can be achieved. For example, Ketmen et al. synthesized reduced graphene oxide-magnetic nanoparticle (rGO-MNP) nanocomposites (Figure 7). Then, they covered the surface of rGO-MNP with polydopamine (PDA). PDA can serve as a cross-linking agent in addition to immobilization platforms. Afterward, the produced rGO-MNP-PDA nanocomposite was blended in polystyrene (PS) at a specific ratio creating the electrospinning polymer solution. PS/rGO-MNP-PDA ESNFs were produced using the electrospinning technique. The developed PS/rGO-MNP-PDA ESNFs were used as an immobilization matrix for anti-CRP to detect CRP in saliva using electrochemical measurements. They indicated that using MNP-modified rGO allowed acquiring better electrochemical signals due to the fast electron transfer ability of rGO and MNP on the electrode surface. The developed PS/rGO-MNP-PDA/anti-CRP immunosensor exhibited a wide linear range between 0.5–100 ng/mL.^21^

Table 3 offers a concise and informative summary of recently developed biosensor systems based on ESNFs with GONM.

**Table 3:** Recently Developed Sensors Using ESNFs with GONM^a^

GONM-ESNFs	analyte	technique	refs
PVP/Chi/rGO	17α-ethinylestradiol	electrochemical	(110)
lignin/PAN/GR	acetaminophen	electrochemical	(109)
Cu-nanoflower@AuNPs-GO	glucose	electrochemical	(111)
PS/GO-APTES	CD36	electrochemical	(42)
GO/PVA/ALP	Hg^2+^, Pb^2+^, Cd^2+^	electrochemical	(112)
rGO-Au/TMC/CNs	glucose	impedimetric paper-based	(113)
GR/PANI/PS	Pb^2+^ Cd^2+^	electrochemical	(114)
RGO-ZnFe_2_O_4_	H_2_S	gas-sensing	(115)
PAN/rGO	17α-ethinylestradiol, estrone, estradiol, and progesterone	impedimetric	(116)

a H_2_O_2_, hydrogen peroxide; PAN, polyacrylonitrile; PVP, polyvinylpyrrolidone; Chi, chitosan; Cu, copper; AuNPs, gold nanoparticles; PVA, poly(vinyl alcohol); GOD, glucose oxidase; ALP, alkaline phosphatase; Hg^2+^, mercury; Pb^2+^, lead; Cd^2+^, cadmium; TMC, trimethyl chitosan; CNs, cellulose nanofibers; PS, polystyrene; GQDs, graphene quantum dots; MWCNT, multiwalled carbon nanotubes; ZnFe_2_O_4_, zinc ferrite; H_2_S, hydrogen sulfide; APTES, (3-aminopropyl)triethoxysilane.

ESNFs-GONM composites are potential candidates for manufacturing and commercializing miniature novel biosensors and flexible/wearable devices that enable point-of-care analysis in the clinical field due to their exclusive structure, outstanding synergy, and excellent conductivity.^117^

### 1.3 Carbon Dots Incorporated ESNFs

**Figure 8:**
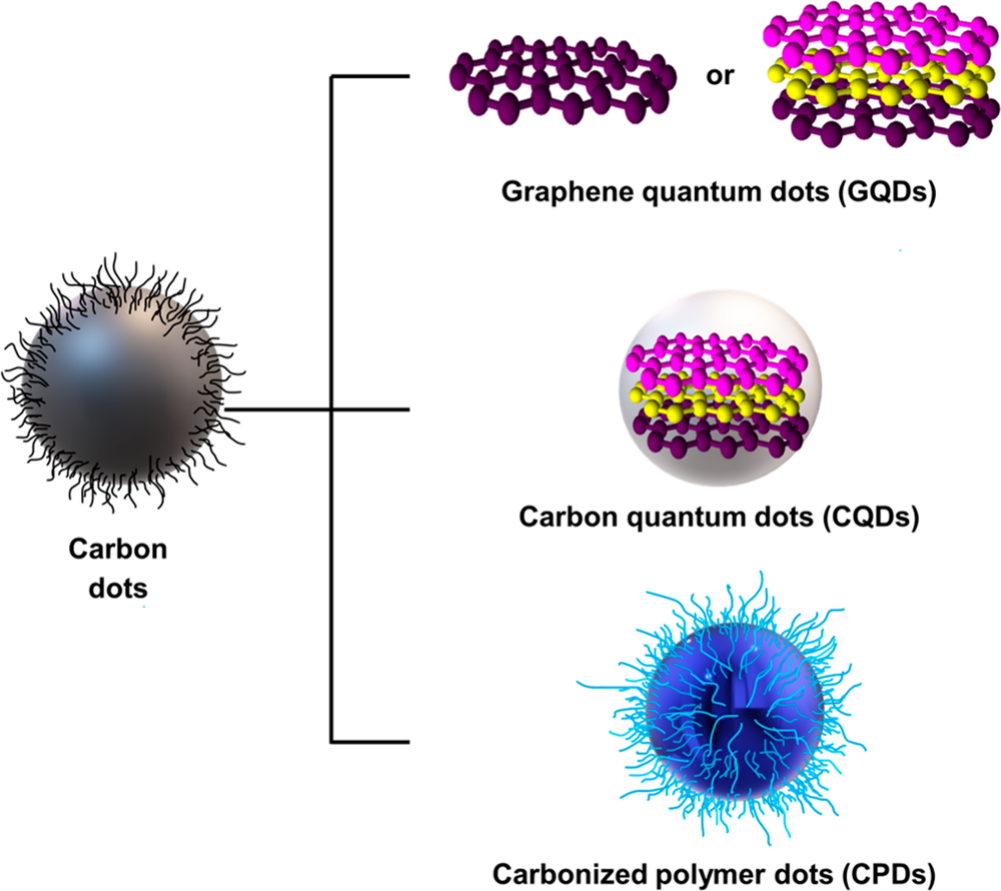
Classification of CDs: GQDs, CQDs, and CPDs.

Carbon dots (CDs), which are carbon-based fluorescent nanomaterials, can be categorized into three types: graphene quantum dots (GQDs), carbon quantum dots (CQDs), and carbonized polymer dots (CPDs) (Figure 8).^118^ CDs are carbon nanoparticles smaller than 10 nm in diameter that exhibit similar physical and chemical properties to graphene such as high chemical stability, broadband optical absorption, suitability for surface modifications, and tunable photoluminescence (PL) properties.^119−123^ Fluorescence in CDs is a result of either the existence of a conjugated π-domain or surface defects generated by surface passivation. The color of CDs’ fluorescence emission is determined by surface groups rather than size, making size modification an inefficient way to control the PL color.^123^ CQDs consist of multiple graphene layers and surface chemical groups with a confinement effect (QCE). Anisotropic GQDs, which include one or a few graphene sheets, have π conjugation on the edge or interlayer defects that provide the quantum confinement and edge effect.^124^ While PL is mainly caused by the QCE in CQDs and GQDs,^125^ CPDs’ optical properties come from the molecular state and cross-link enhanced emission (CEE) effect, unlike GQDs and CQDs.^126^

CQDs and CPDs are usually synthesized through “bottom-up” methods such as the hydrothermal process, microwave-assisted synthesis using small molecules, polymers, or biomass by polymerization, cross-linking, and carbonization.^127,128^ Synthesized CQDs contain carbon, hydrogen, and oxygen. However, functional groups such as carbonyl, hydroxyl, and epoxy can be obtained on the surface by oxidation of CQDs. Moreover, nitrogen, sulfur, and other elements can be easily incorporated into the structures of CQDs using the doping method.^129^ The properties of CQDs can be enhanced by surface functionalization and heteroatom doping. Heteroatom doping can improve the properties of nanomaterials, including electronics, optics, and reactivity, in desired directions.^130^ Notably, there are studies in the literature showing that the optical properties are improved after nitrogen is used as a dopant in graphene-based nanomaterials.^131^ Portable sensing devices could benefit from solid-state arrays of stable CQDs due to their advantages in separation and post-treatment. Therefore, incorporating CQDs into solid matrices may effectively preserve their sensing abilities.^132^ GQDs are a type of CQDs known for their remarkable properties with a crystalline morphology, the structure of graphene lattice, and a thickness of less than 2 nm.^123,129^ GQDs can be synthesized via nanolithography or chemical breakdown of GO.^123^ The fluorescence occurs as the electrons transition between the highest occupied molecular orbital (HOMO) and the lowest unoccupied molecular orbital (LUMO). GQDs of different sizes have varying fluorescence due to the dependence of HOMO–LUMO bandgap on the size of GQDs. As GQDs increase in size (and the energy gap decreases), their emission color shifts from blue to brown in this way.^133^ CQDs have more surface defects and lower crystallinity due to less sp^2^ carbon compared to GQDs, another type of zero-dimensional carbon-based nanomaterials.^134^ Nevertheless, CQDs and GQDs are ideal for binding to redox-active biological substances such as enzymes due to their size and electrical properties.^135^ Studies in the literature focus on preserving GQD fluorescence and sensing ability after immobilization, often through postdeposition or complex encapsulation methods.^127,136,137^ Developing strategies for the one-step integration of GQD onto solid surfaces will contribute to the large-scale and high-yield production of sensing membranes. Electrospinning proves to be a valuable approach to incorporating GQD into filtering membranes. This method offers numerous benefits, including regulating the membrane’s fiber diameter, thickness, and areal weight, resulting in exceptional mechanical strength, porosity, and surface area per unit mass.^138^ The electrospinning technique is a low-cost, reliable method to produce membrane-based sensing platforms. For example, Zhang et al. produced a fluorescence and electrochemical biosensing platform by immobilizing GQD in a nanofibrous membrane through electrospinning water-soluble GQD and poly(vinyl alcohol) to detect H_2_O_2_.^139^ Ratlam et al. developed a biosensor for dopamine detection using PANi/CQDs.^135^ A matrix composed of nanofibers was produced through electrospinning, and the electrochemical measurements were performed with NFs on the fluorine-doped tin oxide-coated glass substrate. The developed biosensor showed low LOD (0.1013 μM) and linear range (10–90 μM) with good sensitivity and selectivity. Studies in the literature have shown that CQDs and GQDs are used instead of CPDs to integrate ESNFs. Table 4 summarizes the recently developed sensors using ESNFs with CQDs and GQDs.

**Table 4:** Recently Developed Sensors Using ESNFs with CQDs and GQDs^a^

CQDs-ESNFs, GQDs-ESNFs	analyte	technique	refs
PANi/CQDs	dopamine	electrochemical and fluorescent	(135)
PCL/CQDs	*S. aureus*	fluorescent	(138)
PVA/GQD ENM	glucose H_2_O_2_	electrochemical and fluorescent	(139)
GQDs-NiO-Au ESNFs/MWCNTs	progesterone	electrochemical	(140)
PVA/citric acid based GQDs	glucose	fluorescent	(141)
PCL-gelatin core-shell/antiCD63/GQD-anti-PSMA	PCa tissue specic cexosomes	sandwich-type immunoassay	(142)
rGO QDs/ZnO hybrid nanofibers	H_2_O_2_ released from cancer and noncancer cells.	electrochemical	(143)
CDs/Fe_3_O_4_	H_2_O_2_ and ascorbic acid	colorimetric detection	(144)
NGQDs@NCNFs	nitrite	electrochemical	(145)

a PANi, polyaniline; mesoSiO_2_, encapsulated mesoporous silica; NiO-Au, nickel oxide-gold; PVA, poly(vinyl alcohol); ENM, electrospun nanofibrous membrane; PCL, polycaprolactone; rGO QDs, reduced graphene oxide quantum dots; PSMA, type II membrane glycoprotein; PCa, prostate-specific antigen; H_2_O_2_, hydrogen peroxide; Fe_3_O_4_, iron oxide; NGQDs, nitrogen-doped graphene quantum dots.

ESNF-CD nanocomposites have proven to be highly promising in their ability to facilitate the production of fluorescence and electrochemical sensors. With their impressive PL feature, these nanomaterials boast excellent potential for various sensing applications.

### 1.4 Nanodiamonds Incorporated ESNFs

Nanodiamonds (NDs), called sp^3^ carbon nanoparticles, are promising nanomaterials in biomedical, sensor development, and drug delivery due to their small size (2–8 nm), high surface areas, and nontoxic and optoelectronic properties.^99,146^ NDs can be synthesized through detonation, pulsed laser ablation, chemical vapor deposition (CVD), or milling of high-pressure-high-temperature (HPHT) microdiamonds. There are studies in the literature in which NDs are incorporated into the structure of ESNFs to increase nanofibers’ mechanical properties and durability.^147−149^ However, ND can cause problems when combined with polymer solutions as they tend to form aggregates. In the electrospinning technique, polymer surface tension and electrostatic attraction that pull the fiber prevent the aggregation of ND. Moreover, solvent evaporation during the fabrication of the fiber effectively prevents the reaggregation of ND. In this way, homogeneously dispersed NDs in the nanofiber can be obtained.^150^ Karami et al. successfully achieved a homogeneous distribution of NDs within the nanofibers even in polymer solutions containing high NDs up to 80% by weight using the electrospinning technique.^151^ It can be concluded that the diameter of the ESNFs obtained by integrating ND into polymer solutions decreases and facilitates electrospinning. However, nanofibers may not be obtained due to increased viscosity in polymer solutions containing ND above a specific ratio. For this reason, the ND ratio in the polymer solution is the factor that affects the diameter and homogeneous distribution of the nanofibers to be obtained.^150^ NDs’ surface can be donated with functional groups such as carboxyl and hydroxyl for conjugation with biological molecules such as enzymes and antibodies using coupling agents (1-ethyl-3-(3-(dimethylamino)propyl)-carbodiimide hydrochloride (EDC) and *N*-hydroxysuccinimide (NHS).^152^ Alshawafi et al. produced ND-incorporated poly(methyl methacrylate) (PMMA) ESNFs and used them as an immobilization matrix for horseradish peroxidase (HRP). They found that the immobilized HRP displayed higher stability and resistance to proteolysis by trypsin than that of soluble enzyme. Thus, they showed the potential application of ND-incorporated PMMA ESNFs in the biosensor field.^152^ Additionally, fluorescent nanodiamonds (FNDs) offer a significant contribution to the future of quantum sensing in biological environments, including thermal and magnetic signals. New surface modification techniques and biocompatible conjugation allow for highly sensitive *in vivo* measurements of static and time-dependent fields. The nitrogen vacancy center (NV center), which is formed by the combination of a nitrogen atom with a vacant diamond lattice site, provides stable luminescent defects. The NV center in diamond forms through ion implantation and annealing. The HPHT method is remarkable for its ability to produce stable luminescent defects under high pressure and temperature conditions.^153^ Optically detected magnetic resonance spectrum (ODMR), which is usually used for magnetic sensing for biological imaging, involves the detection of NV luminescence using sweeping radio frequency signals. Price et al. produced FNDs-contained polylactic-*co*-glycolic acid (PLGA) ESNFs for optical quantum sensing of neural stem cell function. Millisecond temporal resolution and 3.4 μT Hz^–1/2^ sensitivity were achieved for the realization of neural activity using FNDs-PLGA ESNFs.^148^Table 5 summarizes the recently developed sensors using ESNFs with NDs.

**Table 5:** Recently Developed Sensors Using ESNFs with NDs^a^

ESNFs-NDs	analyte	technique	refs
AOx/P(l-Asp)/NDs-CNF/GCE	l-ascorbic acid	electrochemical	(154)
silk-PEO/NDs	temperature-sensing capability of NDs inside the HaCaT cells	fluorescent	(155)
fNDs -PLGA	neural stem cell	optical quantum sensing	(148)

a AOx, ascorbate oxidase; P(l-Asp), poly(l-aspartic acid); CNF, carbon nanofiber; GCE, glassy carbon electrode; PLGA, poly lactic-*co*-glycolic acid; fNDs, fluorescent nanodiamonds.

In the literature review, only a few studies were found that developed biosensors by incorporating nanodiamonds into ESNFs. Modifying sensors with nanodiamonds can create biosensors, as studies have shown. In biosensor applications, nanodiamonds as promising materials can be combined with other nanomaterials, especially ESNFs, to develop high-quality and potential biosensors.

### 1.5 Fullerene Incorporated ESNFs

**Figure 9:**
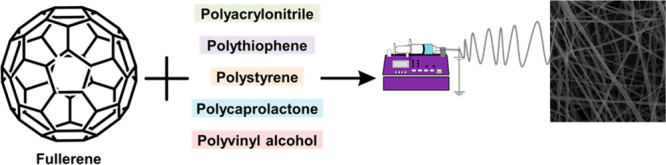
Fullerene incorporated ESNFs.

Due to their electron nature and ease of chemical manipulation, fullerenes are molecular allotropes of carbon that display a variety of fascinating behaviors.^156^ The third carbon allotrope, buckminsterfullerene (C60), was identified by Curl, Kroto, and Smalley in 1985. Fullerene molecules, which can be hollow spheres, ellipses, or tubes, are made of carbon atoms. A schematic representation of the C60 fullerene is given in Figure 9.

Buckyballs are another name for the bucky forms of spherical fullerenes. The fullerene family is a crucial building block because of the diverse chemical behavior made possible by the vast curvature of these hollow spheres’ conjugated-electron systems. They may be used as a medicinal agent due to their carbon cage’s unusual design and broad derivatization range. Since fullerenes are insoluble, interest in their biological uses has increased. The functionalization of fullerenes with hydroxyl and carboxyl groups makes them water-dispersible which is critical for biomedical applications.^157^ The fullerene family, mainly C60, has appealing physical, electrochemical, and photographic capabilities that may be applied in many medical situations.^158^ C60 is a desirable scaffold for drug administration since it can be multifunctionalized, formed, and act as a drug absorbent when compared to the other fullerene derivatives.^157^ Fullerenes can act as a radical scavenger and antioxidant.^159^ Additionally, fullerenes may be employed in energy conversion systems because of their excellent electrochemical stability, small size, unique form, and well-ordered structure.^160^ Fullerene nanofillers have been discovered to improve the physical characteristics of polymers.^161^ The dispersion and miscibility with the polymeric matrix is the primary interaction in the development of the polymer/fullerene nanocomposite.^162^ The fullerene molecules have been altered for this reason to create a physical or chemical interaction with the polymers. The remarkable electrical or semiconducting materials have made the conducting polymer well-known in literature.^163^ The PANi, PPy, polythiophene, and derivative polymeric matrices have been used to build the conducting polymer and fullerene-derived nanocomposite.^164^ Additionally, electrospinning is preferred for obtaining polymer/fullerene nanocomposite nanofibers due to simple equipment, and morphology control.^161^ The nanostructured carbon class member fullerene (C60) has electrocatalytic capabilities that have been described for use in several applications, including as electrochemical sensors and detecting techniques (Table 6. In addition, C60 is used as a mediator between the recognition and the electrode site in electrochemical biosensors because it has inner redox activity.^165^ Partially reduced fullerene-C60-modified electrodes have exhibited excellent working electrodes with properties such as a high electroactive surface area, superior electrical conductivity, and appropriate biocompatibility.^166^ Zu et al. reported that combining KOH-etching and pyrolysis of C60 powder can produce pentagon defect-rich porous carbon. This carbon shows superior oxygen reduction reaction activity compared to the graphite-derived carbon matrix prepared by the same procedure.^167^ Liu et al. developed EP composite coatings enhanced with varied concentrations of fullerene C60 or FG on a cast iron substrate to evaluate the effect of the filler shape on the tribological and anticorrosion performances. C60 and FFG had (3-aminopropyl)triethoxysilane chemically bonded onto their surfaces to improve their dispersion and compatibility with the EP matrix. Fullerene C60 or functionalized graphene nanofillers added to the EP matrix produced better tribological results.^168^ By employing a liquid–liquid interfacial precipitation technique and C60-saturated solutions in *N*-methyl-2-pyrrolidone and isopropyl alcohol, Qu et al. effectively produced fullerene C60 nanofibers. The solvents must include a nitrogen atom, which contains a solitary electron pair and enables the solvents to act as electron donors to produce fullerene C60 nanofibers. A critical factor in creating fullerene C60 nanofibers is the development of charge transfer adducts between C60 and fluids with a single electron pair.^169^ A fullerene nanofiber based on supramolecular pentapeptides was created by Insuasty et al. The b-sheets and -interactions between the C60 molecules stabilize the nanofibers.^170^ It was discovered that when fullerene was enclosed in nanofibers, the contact angle was dramatically reduced. It is common practice to analyze bond types and identify unidentified chemicals using FTIR analysis.^171^

**Table 6:** Developed Sensors Using ESNFs with Fullerene^a^

ESNFs- Fullerene	analyte	technique	refs
PLGA/C60	d-gluconic acid	piezoelectric	(173)
PAN/C60/GOD	glucose	electrochemical	(174)

a PLGA, poly(dl-lactide-*co*-glycolide); PAN, polyacrylonitrile; GOD, glucose oxidase.

Under typical environmental circumstances, it is possible to produce fullerene nanofibers, which are fine needle-like crystals that contain fullerene molecules.^172^ In several study domains, spherical fullerenes, mainly C60, the most stable type of fullerene, are highly favored. Because of their various surface characteristics (energies), or “willingness” to be well distributed in multiple solvents, numerous derivatives of C60 are employed. Most people are aware that pure C60 is a hydrophobic substance. As mentioned before, the hydrophilic −OH groups on the fullerene surface or the fullerene oxide derivative enable the creation of its improved dispersion in water.

Although fullerene-based nanomaterials arouse interest due to their unique precise molecular structure for monitoring the catalytic process, the literature review revealed very few studies on integrating fullerene-based nanofibers into biosensors. Therefore, the role of fullerene in electrochemical catalysts needs to be further investigated.

## 2 Conclusions and Prospective

Nanomaterials are used in many fields of biotechnology such as biosensors, tissue engineering, and environmental applications. In biosensor preparation, nanomaterials are attractive structures due to their exclusive properties such as large surface, high stability, and special optical and analytical performances, etc. Among them, ESNFs are great materials to fabricate biosensors. ESNFs are produced from various natural and synthetic polymers or both. By the addition of carbon nanomaterials to nanofibers architecture, the synergic effects of both can be obtained. Carbon-based ESNFs made from nontoxic polymers can create wearable sensor systems due to their flexibility and biocompatibility, in addition to electrochemical biosensors. Thus, they have a high application potential to design innovative and effective noninvasive detection devices. Their functional surface groups provide covalent conjugation of biomolecules onto their surface to prepare biosensors with higher stability. In summary, integrating carbon-based ESNFs into biosensors can create effective devices for biosensing and have commercial applications for point-of-care (POC) diagnosis.
